# 

**Published:** 1879-07

**Authors:** 


### CONTENTS.

ORIGINAL.

ART. I.—Portions of the Address by Prof. Thomas F. Rochester, M. D., of Buffalo. Chairman of the Section on Practical Medicine before Am. Med. Association, 1879,.. 441

CORRESPONDENCE.

Letter from E. V. Stoddard, Berlin............ 456
Letter from Surgeon-General J. B. Hamilton to Medical Officers,....... ...... .......... 459

MISCELLANEOUS.

Letter from Vienna—Clinics for Skin Diseases and Syphilis,—Billroth's Operation, &c.,.. 460
Intravenous Injection of Milk in Bright's Disease,.................................. 464
The Identity of the Cells in Fluid from Cystic Tumors of the neck, scrotum, etc., with the so-called Ovarian Corpuscle, or Drysdale Cell,.................................. 468
Wisconsin Medical Act,.................. .. 469

EDITORIAL.

Remarkable petition for Insane Asylum investigation,...... .......... ............ .. 470
Completion of Vol. XVIII. and the future of the Journal,... ........... .............. 471
Office Card,..................................... 471
Death of Dr. James Ives,............. ....... 471
BOOKS REVIEWED—Spermatorrhœa, by Roberts Bartholow, .. ........ .................. 471
Tabular Handbook of Auscultation and Percussion, by Herbert C. Clapp, A. M., M. D., Boston,........ ....................... 472
National Dispensatory, by Alfred Stillé and John Marsch......... ..................... 473
A Guide to the Qualitative and Quantitative Analysis of the Urine,.... .............. 473
Books Received,....................... ........ 474

